# Risk factors and associated outcomes of ventilator-associated events developed in 28 days among sepsis patients admitted to intensive care unit

**DOI:** 10.1038/s41598-020-69731-3

**Published:** 2020-07-29

**Authors:** Wen-Feng Fang, Ying-Tang Fang, Chi-Han Huang, Yu-Mu Chen, Ya-Chun Chang, Chiung-Yu Lin, Kai-Yin Hung, Ya-Ting Chang, Hung-Cheng Chen, Kuo-Tung Huang, Huang-Chih Chang, Yun-Che Chen, Yi-Hsi Wang, Chin-Chou Wang, Meng-Chih Lin

**Affiliations:** 1 Division of Pulmonary and Critical Care Medicine, Department of Internal Medicine, Kaohsiung Chang Gung Memorial Hospital, Chang Gung University College of Medicine, 123 Ta-Pei Rd, Niao-Sung Dist., Kaohsiung, 833 Taiwan; 2 Department of Respiratory Therapy, Kaohsiung Chang Gung Memorial Hospital, Chang Gung University College of Medicine, Kaohsiung, Taiwan; 3 Department of Respiratory Care, Chang Gung University of Science and Technology, Chiayi, Taiwan; 4 Graduate Institute of Clinical Medical Sciences, Chang Gung University, Taoyuan, Taiwan; 5 Department of Nutritional Therapy, Kaohsiung Chang Gung Memorial Hospital, Kaohsiung, Taiwan

**Keywords:** Cytokines, Predictive markers

## Abstract

We hypothesized that Ventilator-Associated Event (VAE) within 28 days upon admission to medical intensive care units (ICUs) can be a predictor for poor outcomes in sepsis patients. We aimed to determine the risk factors and associated outcomes of VAE. A total of 453 consecutive mechanically ventilated (MV) sepsis patients were enrolled. Of them, 136 patients had immune profile study. Early VAE (< 7-day MV, n = 33) was associated with a higher mortality (90 days: 81.8% vs. 23.0% [non-VAE], P < 0.01), while late VAE (developed between 7 and 28 days, n = 85) was associated with longer MV day (43.8 days vs. 23.3 days [non-VAE], P < 0.05). The 90-day Kaplan–Meier survival curves showed three lines that separate the groups (non-VAE, early VAE, and late VAE). Cox regression models with time-varying coefficient covariates (adjusted for the number of days from intubation to VAE development) confirmed that VAE which occurred within 28 days upon admission to the medical ICUs can be associated with higher 90-day mortality. The risk factors for VAE development include impaired immune response (lower human leukocyte antigen D-related expression, higher interleukin-10 expression) and sepsis progression with elevated SOFA score (especially in coagulation sub-score).

## Introduction

Ventilator-associated event (VAE)^1,2^ was proposed to overcome the limitations of only focusing on ventilator-associated pneumonia (VAP)^3^ surveillance in mechanically ventilated (MV) patients in the context of surveying quality improvement. The limitations of previous VAP surveillance, which is proven to be neither sensitive nor specific^4^, hindered its application in quality improvement programs^5^. Although VAE surveillance was a promising early warning tool for VAP prevention^6^, VAP prevention bundle compliance was not associated with a reduced risk of VAE^7^. VAEs are defined as respiratory deterioration after a period of improved or stable gas exchange. The definition of VAE shifted the focus of surveillance from pneumonia to all conditions caused by mechanical ventilation including infectious or noninfectious conditions. The purpose was to expand the scope of surveillance to include multiple serious complications in ventilated patients, not just pneumonia, as well as to make surveillance more objective, efficient, and suitable for electronic implementation.

Sepsis patients developed life-threatening organ dysfunctions (e.g., acute respiratory failure) caused by dysregulated host immune response to infection^8–10^. An increase in sequential organ failure assessment (SOFA) score (including respiratory and other five sub-scores) has prognostic accuracy for in-hospital mortality in sepsis patients in the intensive care units (ICUs)^11^. Since mechanical ventilator support and aggressive fluid resuscitation are usually performed as sepsis management, sepsis patients are vulnerable to VAE development due to fluid overload^12^, ventilator-associated lung injury, VAP, and multiple organ dysfunction. The association between sepsis and VAE is complicated. Although several studies have tested the prognostic accuracy of VAE^13–15^ in the medical and surgical ICUs such as for trauma patients, only a few studies have been conducted among sepsis patients. Whether the presence of early VAE or late VAE influences the outcomes of sepsis patients remains unclear.

We hypothesize that VAE can be a predictor for poor outcomes (90-day mortality as the main outcome) in sepsis patients and is associated with impaired immune profile and abnormal values in serial SOFA sub-scores. The early onset of VAE (within 7 days upon ICU admission) may reflect the progression of sepsis in the first week, and late VAE (developed between day 7 and day 28) may indicate poor recovery from sepsis. The characteristics and outcomes of non-VAE, early VAE, and late VAE may be different. This study aimed to determine whether sepsis patients who develop VAEs within 28 days had poorer outcomes, regardless of early or late onset. It also aimed to investigate the predictors for early and late VAEs.

## Methods

### Setting

The study was conducted in three medical ICUs (total 34 beds) from August 2013 to January 2016 at Kaohsiung Chang Gung Memorial Hospital, a 2,700-bed tertiary hospital in Southern Taiwan.

### Study design

The retrospective analysis study is a part of integrated research programs, consisting of prospective observational study (immune profile study) and retrospective medical record review (e.g., consecutive mechanically ventilated sepsis patients), that investigate the clinical factors, biomarkers, and immune response in predicting outcomes in sepsis patients^10,16–18^. All patients who met the Sepsis-3 criteria^8^ and admitted to the medical ICUs with invasive mechanical ventilation support were screened. The enrolled patients with sepsis were admitted to the ICU before development of VAE. Patients receiving noninvasive mechanical ventilation or extracorporeal membrane oxygenation use were excluded. To meet the VAE criteria, we also excluded those patients who never achieved stable ventilator setting after intubation or died within 3 days of mechanical ventilation initiation.

The study was approved by the Institutional Review Board of Chang Gung Memorial Hospital. We confirmed that all methods were performed in accordance with the relevant guidelines and regulations. For the patients who prospectively participated in immune profiling and cytokine analysis, patients or their surrogates had signed the written informed consent. The requirements to obtain informed consent for the retrospective analysis part of the study were waived by Institutional Review Board.

### Definition and criteria for VAE: early VAE and late VAE

The following three categories of VAE were identified: (1) ventilator-associated condition (VAC), (2) infection-related ventilator-associated complication (IVAC), and (3) possible VAP^19^. VAC is defined as an increase of at least 3 cmH_2_O in daily minimum positive end expiratory pressure (PEEP) or an increase of at least 20 points in daily minimum fraction of inspired oxygen (FiO_2_) over baseline for at least 2 days, after at least 2 days of stable or decreasing PEEP or FiO_2_^20^. IVAC belongs to the subset of VAC that may be infection related. VAP was defined as the presence of purulent secretions or positive pulmonary cultures. The culture specimens utilized for the testing of pneumonia organisms include sputum, tracheal aspirate, bronchoalveolar lavage fluid, pleural effusion, blood, and urine for *Legionellae* antigen test or *Streptococcus pneumoniae* antigen test^21^. Early VAE (within 7 days) and late VAE (between day 7 and day 28) were defined as the period from the initiation of mechanical ventilation to the onset of VAC. Patients who had an early VAE were not included in the late VAE model. Long-term ventilator dependence was defined as the need for mechanical ventilation for more than 6 h per day for more than 21 days^22^.

### Data collection

**Figure 1:**
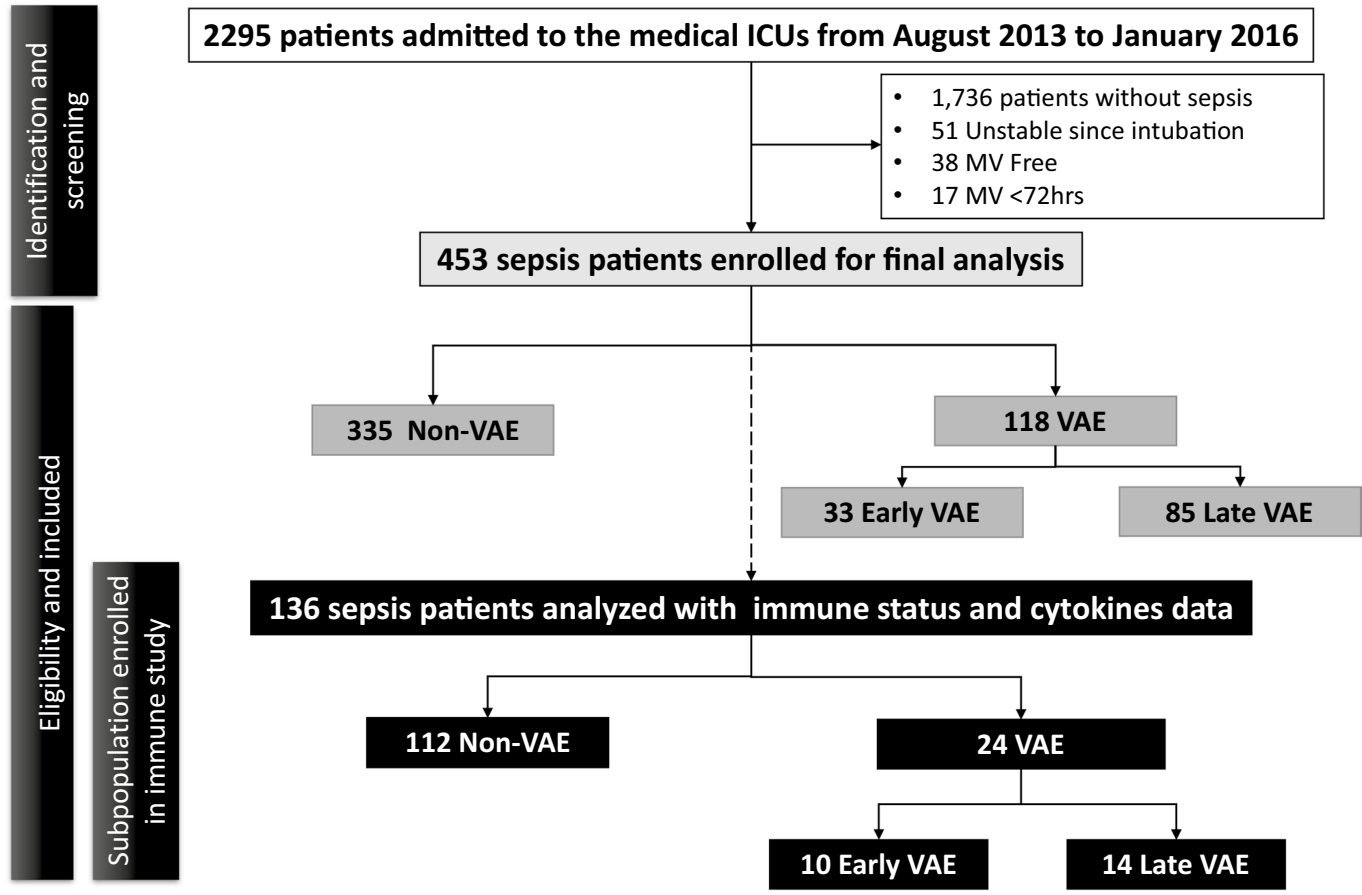
Study flowchart.

Clinical data were retrieved from the medical records including SOFA score^11,23,24^, Acute Physiology and Chronic Health Evaluation II (APACHE II) score^25,26^, Charlson Comorbidity Index, and underlying comorbidities and other clinical factors that were possibly related to the occurrence of the condition. The factors for possible VAP were utilized for VAE surveillance^1,27,28^. Moreover, 136 patients had been enrolled in immune status and cytokine study with blood sampling tests performed based on the protocol on days 1, 3, and 7 during ICU admission (Fig. 1).

### Immune status and cytokine study

Plasma and peripheral blood mononuclear cell preparation, measurements of human leukocyte antigen D-related (HLA-DR) monocyte expression, and cytokine levels have been described in our previous studies^9, 10^.

### Statistical methods

Patient demographics, clinical characteristics, and outcomes were expressed using frequency and percentage for categorical variables and mean ± standard deviation or median (interquartile range, IQR) for continuous variables (Table 1). The differences between the non-VAE group and VAE group were analyzed using the Student’s t-test or Mann–Whitney U test, as appropriate, for continuous variables or the chi-square test for categorical variables. Comparison analyses among the non-VAE, early VAE, and late VAE groups were performed using the Pearson chi-square and one-way analysis of variance as appropriate. Pairwise comparisons were conducted using the analysis of variance (ANOVA) with adjustment for multiple comparisons utilizing Tukey's range test for post hoc comparisons (Table 2). Kruskal–Wallis test was used as a non-parametric alternative to the ANOVA for non-normally distributed continuous variables. Clinical parameters [e.g., baseline characteristics, SOFA sub-scores, laboratory data, oxygenation index, lung mechanics, and input/output (I/O) fluid balance on days 1, 3, and 7; details in Table 1 or e-Tables 1, and 2(a, b)] thought to be predictors of VAE development (early VAE and late VAE, respectively) were analyzed using a univariable regression analysis. The factors that were found to be statistically significant (P < 0.1) in the univariable regression analysis were then retained and included in the multivariable logistic regression model using backward elimination of logistic regression analysis to determine whether they remained predictive for early VAE (Table 3) or late VAE (Table 4) development. Patients who have been on invasive ventilation for at least 7 days are at risk for late VAE. The analytical population for late VAE was limited to those with at least 7 days of mechanical ventilation and was included in the multivariable logistic regression model. To assess the survival outcome between groups, Kaplan–Meier survival curves were constructed; comparison between groups was performed using the log-rank test. The mortality hazard ratios between groups were also compared using Cox regression models with time-varying coefficient or fixed (non-time-dependent) covariates. The number of days from intubation to VAE development was then adjusted. Statistical significance was set at a two-sided P value of < 0.05. All data were analyzed using the Statistical Package for the Social Sciences software version 22.0 (IBM Corp., Armonk, NY, USA).

**Table 1:** Baseline characteristics of 453 patients with sepsis and comparison of the non-VAE and VAE groups.

Demographics characteristics	Total (N = 453)	Non-VAE (N = 335)	VAE (N = 118)	P
Age (years)	67.8 ± 14.8	68.1 ± 15.2	67.3 ± 13.9	0.641
BMI (kg/m^2^)	22.6 ± 4.9	22.8 ± 5.0	22.2 ± 4.8	0.281
Sex, male (%)	269 (59.4)	196 (58.5)	73 (61.9)	0.523
APACHE II score	24.8 ± 8.5	25.1 ± 8.7	24.2 ± 8.1	0.305
CURB-65	2.6 ± 0.9	2.6 ± 0.9	2.7 ± 0.9	0.529
PSI	135.7 ± 34.2	134.1 ± 34.9	140.2 ± 32.1	0.096
**Site of suspected infection, N (%)**				
Pulmonary	273 (60.3)	201 (60.0)	72 (61.0)	0.846
Intra-abdominal	34 (7.5)	23 (6.9)	11 (9.3)	0.384
Urinary tract	130 (28.7)	103 (30.7)	27 (22.9)	0.104
Bacteremia	41 (9.1)	32 (9.6)	9 (7.6)	0.531
Unidentified infection	34 (7.5)	27 (8.1)	7 (5.9)	0.451
Charlson Comorbidity Index	2.6 ± 1.8	2.4 ± 1.7	3.2 ± 2.2	0.001
Coronary artery disease	114 (25.2)	84 (25.1)	30 (25.4)	0.940
Hypertension	255 (56.3)	197 (58.8)	58 (49.2)	0.069
COPD	67 (14.8)	52 (15.5)	15 (12.7)	0.460
Cancer	102 (22.7)	59 (17.8)	43 (36.8)	< 0.001
Chronic liver disease	58 (12.8)	39 (11.6)	19 (16.)	0.212
Diabetes mellitus	211 (46.6)	164 (49.0)	47 (39.8)	0.087
History of stroke	99 (21.9)	76 (22.7)	2 (19.5)	0.470
Chronic kidney disease	139 (30.7)	104 (31.0)	35 (29.7)	0.779

Categorical data are expressed as proportions and compared using the Pearson chi-square test. Differences between the non-VAE and VAE groups were analyzed using the Student’s t-test for continuous variables. CURB65 and PSI is applicable only to patients admitted with CAP.

*BMI* body mass index, *APACHE II* Acute Physiology and Chronic Health Evaluation score, *PSI* pneumonia severity index, *COPD* Chronic obstructive pulmonary disease.

**Table 2:** Effects of non-VAE, VAE, early VAE, and late VAE on clinical primary and secondary outcomes in sepsis patients.

Items	Total	Non-VAE	VAE	P	Early VAE	Late VAE	P^†^	*P*^§^
	(N = 453)	(N = 335)	(N = 118)		(N = 33)	(N = 85)		
VAE, n (%)	118 (26.0)	–	–		–	–		
Ventilator days to event, d (IQR)	13 (7–24)	–	13 (7–24)		5 (4–6) **	17 (12–28)^**##**^	< 0.001	< 0.001
Ventilator dependence, n (%)	144 (31.8)	76 (22.7)	68 (57.6)	< 0.001	10 (30.3)	58 (68.2)^**##**^	< 0.001	< 0.001
Ventilator duration, d ± SD	26.7 ± 59.4	23.3 ± 63.0	36.4 ± 46.8	0.040	17.2 ± 16.3	43.8 ± 52.4^**#**^	0.011	< 0.001
ICU LOS, d ± SD	14.5 ± 10.0	12.6 ± 7.5	20.1 ± 13.7	< 0.001	12.8 ± 9.1	22.8 ± 14.1^**##**^	< 0.001	< 0.001
Hospital LOS, d ± SD	35.5 ± 29.1	35.4 ± 26.8	36.1 ± 35.2	0.841	20.9 ± 19.6*	41.9 ± 38.1	0.002	0.003
7-day mortality, n (%)	21 (4.6)	10 (3)	11 (9.3)	0.005	9 (27.3) **	2 (2.4)	< 0.001	< 0.001
14-day mortality, n (%)	47 (10.4)	18 (5.4)	29 (24.6)	< 0.001	17 (51.5) **	12 (14.1)^**#**^	< 0.001	< 0.001
28-day mortality, n (%)	91 (20.1)	38 (11.3)	53 (44.9)	< 0.001	23 (69.7) **	30 (35.3)^**##**^	< 0.001	0.001
90-day mortality, n (%)	170 (37.5)	77 (23.0)	93 (78.8)	< 0.001	27 (81.8) **	66 (77.6)^**##**^	< 0.001	0.992
ICU mortality, n (%)	85 (18.8)	25 (7.5)	60 (50.8)	< 0.001	23 (69.7) **	37 (43.5)^**##**^	< 0.001	0.011
Hospital mortality, n (%)	173 (38.2)	76 (22.7)	97 (82.2)	< 0.001	27 (81.8) **	70 (82.4)^**##**^	< 0.001	0.946

**P**^**†**^: Comparison analyses among the non-VAE, early VAE, and late VAE groups using one-way analysis of variance (ANOVA).

The pairwise comparisons were conducted using the ANOVA with adjustment for multiple comparisons utilizing Tukey's range test for post hoc comparisons or the chi-square test for categorical variables.

**P**^§^: between the early VAE and late VAE.

*P < 0.05, **P < 0.01 between the non VAE and early VAE.

^**#**^P < 0.05, ^**##**^: P < 0.01 between the non-VAE and late VAE.

**Table 3:** Logistical regression to determine sepsis patients with early VAEs.

Characteristics	Multivariable (backward LR)	
	OR (95% CI)	P
Charlson Comorbidity Index	0.811 (0.503–1.307)	0.389
Cancer	2.116 (0.248–18.068)	0.493
**Day 1**		
Coagulation sub-score	0.623 (0.353–1.101)	0.103
**Day 3**		
Respiration sub-score	1.325 (0.813–2.160)	0.258
Coagulation sub-score	1.995 (1.197–3.327)	0.008*
Liver sub-score	1.686 (0.959–2.963)	0.069
CNS sub-score	1.395 (0.826–2.356)	0.213
Renal sub-score	0.936 (0.658–1.331)	0.713

*P < 0.05.

**Table 4:** Logistical regression to predict sepsis in patients with late VAEs.

Characteristics	Multivariable (backward LR)	
	OR (95% CI)	P
**Day 3**		
Respiration sub-score	0.373 (0.141–0.984)	0.046*
Coagulation sub-score	1.841 (1.055–3.210)	0.032*
Renal sub-score	0.759 (0.483–1.192)	0.231
Oxygenation index	1.125 (0.979–1.293)	0.097
I/O, fluid balance	1.001 (1.000–1.001)	0.065
**Day 7**		
Respiration sub-score	2.065 (0.814–5.240)	0.127
CV sub-score	3.419 (1.431–8.167)	0.006*
CNS sub-score	1.389 (0.731–2.641)	0.316
Red blood cells (10^6^/μL)	0.283 (0.102–0.783)	0.015*
C-reactive protein (mg/L)	0.994 (0.984–1.005)	0.285
Lactate (mmol/L)	0.944 (0.872–1.021)	0.152
Oxygenation index	0.907 (0.792–1.038)	0.156
Resistance	1.127 (1.007–1.261)	0.037*
Compliance	0.982 (0.962–1.003)	0.096

*P < 0.05.

### Ethics approval and consent to participate

The study was approved by the Institutional Review Board of Chang Gung Memorial Hospital. We confirmed that all methods were performed in accordance with the relevant guidelines and regulations. For the patients who prospectively participated in immune profiling and cytokine analysis, patients or their surrogates had signed the written informed consent. The requirements to obtain informed consent for the retrospective analysis part of the study were waived by Institutional Review Board.

## Results

A total of 453 sepsis patients were enrolled for analysis. Of them, 136 patients had immune profile study (Fig. 1). A total of 118 sepsis patients (26%) developed VAE after initial respiratory stabilization following sepsis treatment (Table 1). There were no notable differences between the VAE group and non-VAE group in terms of age, gender, body mass index, APACHE II score, and site of suspected infection. Their pneumonia severity scores^21^ (PSI and CURB65) were also comparable. Although more patients with VAE had cancer (VAE vs. non-VAE = 36% vs. 17%, P < 0.001) and higher Charlson Comorbidity Index (3.2 ± 2.2 vs. 2.4 ± 1.7, P = 0.001), the phenomenon disappeared in the multivariable analysis.

### Sepsis patients who developed VAEs with poorer outcomes

Patients in the VAE group (compared to non-VAE) have a longer ICU length of stay (LOS) (20.1 ± 13.7 days vs. 12.6 ± 7.5 days, P < 0.001); longer duration of mechanical ventilation (36.4 ± 46.8 days vs. 23.3 ± 63.0 days, P = 0.040); higher 7-day, 14-day, 28-day, and 90-day ICU and hospital mortality (Table 2, e-Fig. 1); and higher ventilator dependence (57% vs. 22.7%, P < 0.001) (Table 2).

### Different outcomes and serial clinical variables among the non-VAE, early VAE, and late VAE groups

**Figure 2:**
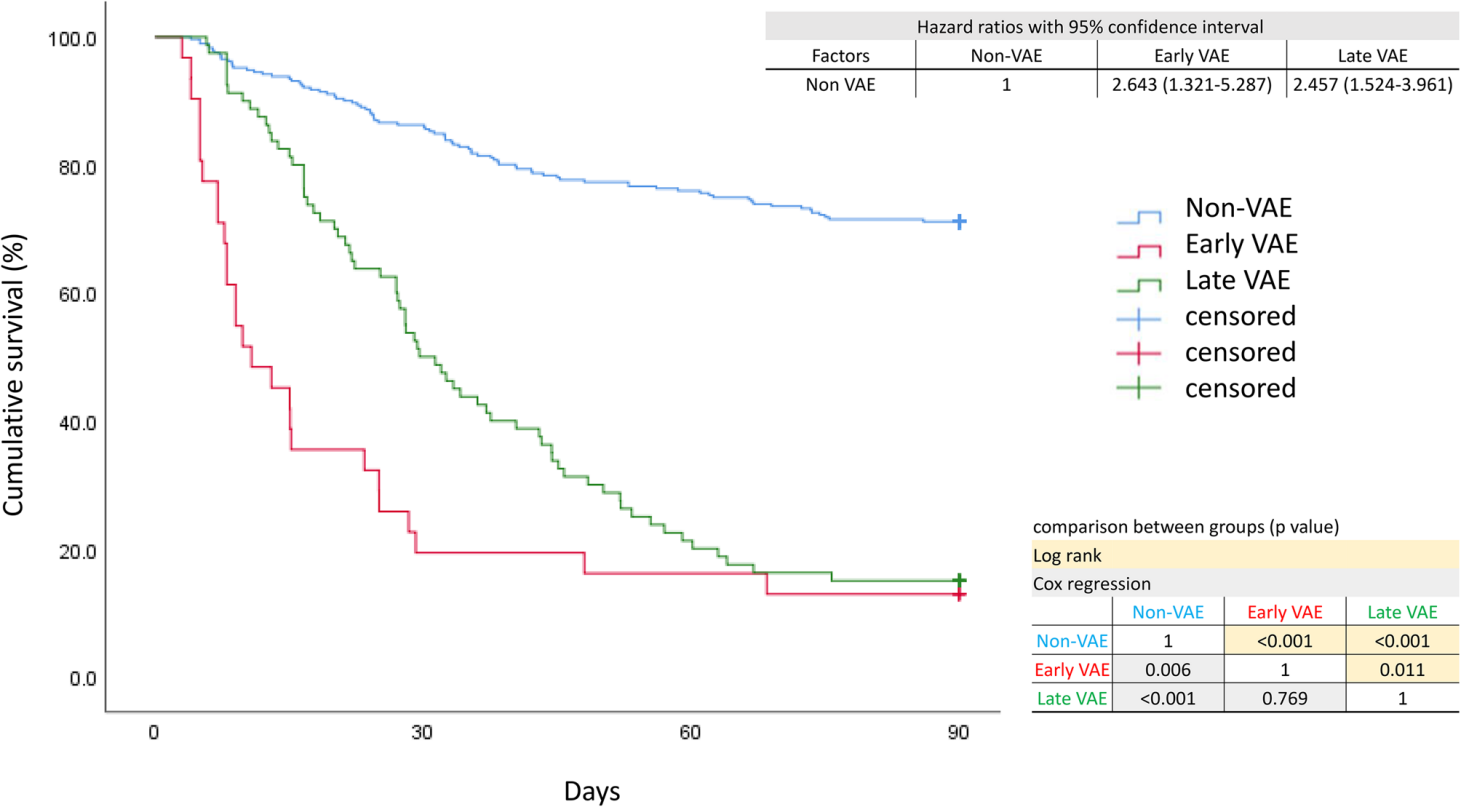
Ninety-day survival curves of the non-VAE, early VAE, and VAE groups. Kaplan–Meier estimates of 90-day survival according to stratification of the three groups. Hazard ratios between groups using Cox regression models with time-varying coefficient were shown. The number of days from intubation to VAE development was adjusted.

The incidence of all VAEs in our study was 26%; patients developed the VAE within a median of 13 days (IQR 7–24) (Table 2). Patients developed the early VAE within a median of 5 days (IQR 4–6) and the late VAE within a median of 17 days (IQR 12–28). The outcomes of the late VAE group were poorer than those of the early VAE group in terms of ventilator dependence, total ventilation days, LOS, and hospital LOS (Table 2). However, early VAE was a good predictor of poorer 7-day, 14-day, 28-day, and ICU mortality than late VAE. The 90-day mortality and hospital mortality were comparable between early VAE and late VAE (Table 2). Kaplan–Meier survival curves showed three lines that separates the non-VAE, early VAE, and late VAE groups (Fig. 2). However, the early VAE and late VAE group showed comparable mortality hazard ratios in the 90-day Cox regression model with time-varying coefficient (time to VAE development after intubation).

E-Table 2(a) and (b) illustrates the clinical characteristics of the non-VAE, early VAE, and late VAE groups in different time points (day 1, day 3, and day 7).

### Predictor for early VAE

No statistically significant differences were observed between the VAE group and non-VAE group in terms of demographic characteristics except for the presence of underlying cancer and poorer Charlson Comorbidity Index in the VAE group (e-Table 1). The fluid balance of the VAE group on day 1 was not significantly different compared with that of the non-VAE group (e-Table 2(a)). However, cumulative positive fluid balance was noted in the early VAE group (e-Table 3). From the analysis on the serial SOFA sub-scores and laboratory data, we found that patients who developed early VAE had poorer coagulation sub-score on day 1 and elevated serum lactate level and SOFA score (including coagulation, liver, and cardiovascular [CV] sub-scores) on day 3 (e-Table 2(a), (b)). Logistical regression yields that the coagulation sub-score on day 3 can predict the development of early VAE (Table 3). The SOFA score has been shown to decrease over time in the non-VAE group (e-Table 4).

### Predictors for late VAE

The presence of underlying cancer and poorer Charlson Comorbidity Index were significantly different between the late VAE group and non-VAE group (e-Table 1). E-Table 2(a) and (b) shows the different variables between patients with late VAE and those without VAE. Cumulative positive fluid balance was noted in the late VAE group (e-Table 3). Table 4 presents some of the characteristics of the late VAE. Multivariable logistic regression yielded that poor coagulation sub-score on day 3, poor CV sub-score on day 7, and increased airway resistance on day 7 were predictors of late VAE development. On the contrary, respiration sub-score on day 3 and red blood cell (RBC) count on day 7 were negative predictors of late VAE.

### Immune status and cytokine expression characteristics of patients with early VAE and late VAE

No difference was observed in the 90-day mortality between patients who had immune data for this analysis and those who did not have the data. They had similar survival curves regardless of VAE status (e-Fig. 2).

In this subpopulation with immune status and cytokine studies (Table 5), the early VAE group had lower HLA-DR expression (76.6 ± 21.1% vs. 87.3 ± 15.0%, P = 0.051) on day 1, while the late VAE group had lower HLA-DR expression (80.8 ± 20.6% vs. 94.3 ± 8.0%, P < 0.05) on day 7. The late VAE group had significantly higher anti-inflammatory cytokine (interleukin-10 [IL-10]) on day 1 and day 7 compared with the non-VAE group. This finding suggests that the patients were immunosuppressed prior to the appearance of VAE. However, the late VAE group showed increased in granulocyte-colony stimulating factor (G-CSF), interleukin-6 (IL-6), and tumor necrosis factor alpha (TNF-α) on day 3. On day 7, the IL-6 concentration in the late VAE group remained high compared with that in the non-VAE group. There were differences in the immune profiles of VAE and non-VAE groups.

**Table 5:** Cytokine parameters.

Day 1	Non-VAE (N = 112)	Early VAE (N = 10)	Late VAE (N = 14)	P^†^	P^§^
HLA-DR expression (%)	87.3 ± 15.0	76.6 ± 21.1	83.9 ± 16.8	0.051	0.285
G-CSF (pg/mL)	788.9 ± 266.1	137.9 ± 95.1	2,608.9 ± 5,463.2	0.099	0.585
IL-10 (pg/mL)	56.4 ± 126.0	311.8 ± 900.2	176.7 ± 293.4^#^	0.007	0.042
IL-6 (pg/mL)	140.4 ± 285.9	201.5 ± 337.2	1,469.7 ± 4,195.9	0.076	0.192
TNF-α (pg/mL)	48.6 ± 49.9	65.9 ± 94.2	149.6 ± 322.2	0.077	0.122

Day 3	Non-VAE (N = 109)	Early VAE (N = 9)	Late VAE (N = 13)	P^†^	P^§^
HLA-DR expression (%)	89.3 ± 13.4	90.7 ± 7.3	83.3 ± 17.7	0.153	0.431
G-CSF (pg/mL)	194.6 ± 646.4	119.4 ± 191.9	2,347.4 ± 5,030.5^#^	0.027	0.051
IL-10 (pg/mL)	35.9 ± 102.5	213.9 ± 623.3	87.3 ± 93.5	0.009	0.021^§§^
IL-6 (pg/mL)	73.8 ± 175.3	390.3 ± 930.4	270.7 ± 665.8^#^	0.005	0.043^§§^
TNF-α (pg/mL)	39.2 ± 36.3	81.7 ± 177.8	87.7 ± 137.3 ^#^	0.029	0.011

Day 7	Non-VAE (N = 98)	Early VAE (N = 6)	Late VAE (N = 12)	P^†^	P^§^
HLA-DR expression (%)	94.3 ± 8.0	97.4 ± 2.2	80.8 ± 20.6^#^	0.003	0.005^§§^
G-CSF (pg/mL)	100.2 ± 226.2	28.6 ± 27.2	71.2 ± 52.7	0.174	0.102
IL-10 (pg/mL)	45.2 ± 164.0	7.4 ± 9.3	67.7 ± 77.7^#^	0.003	0.002^§§^
IL-6 (pg/mL)	45.6 ± 101.8	19.4 ± 27.4	226.0 ± 445.0^#^	0.002	0.002^§§^
TNF-α (pg/mL)	36.4 ± 34.8	19.0 ± 8.6	51.7 ± 26.7	0.006	0.052

**P**^**†**^: Comparison analyses among the non-VAE, early VAE, and late VAE groups using Kruskal–Wallis as a non-parametric alternative to the analysis of variance for non-normally distributed continuous variables.

Post hoc pairwise comparisons.

*P < 0.05, between the non VAE and early VAE.

^**#**^P < 0.05, between the non-VAE and late VAE.

^*§*^P < 0.05, between early and late VAE.

**P**^**§**^: Comparison analyses between the early VAE and late VAE groups using the Mann–Whitney U test.

## Discussion

Our study shows that VAE within 28 days of ICU admission can be a simple predictor of poorer outcomes (mortality and ventilator dependency) for sepsis patients with initially stable respiratory condition after treatment. The notion of “early VAE” vs “late VAE” is novel since it is a concept borrowed from VAP and not hitherto applied to VAE. The interaction between sepsis and VAE before day 7 may be more complicated. Many sepsis studies considered 28-day all-cause mortality as the primary outcome measure. We suggested that VAE that developed after day 28 was less likely to interact with sepsis. Therefore, late VAE was considered respiratory deterioration developed between day 7 and day 28. The patients who did not develop VAE within 28 days were stratified as non-VAE. The characteristics and outcomes of non-VAE, early VAE, and late VAE were different.

VAE development might be related either with worsening of the initial episode of sepsis or a with a new episode. The development of VAE is associated with patients’ impaired immune profile during sepsis. Before the patients developed VAE, bigger positive cumulative fluid balance, abnormal SOFA score and sub-scores, and other risk factors have already been identified. Changes in SOFA sub-scores vary by VAE status (e-Table 4 [see Additional file 1]). We also demonstrated the different effects of early and late VAE on sepsis patients’ outcomes.

VAP is associated with a longer ICU length of stay, longer duration of mechanical ventilation, and higher mortality^29^. Therefore, VAP surveillance was initiated, and VAP prevention bundle care was encouraged. To enhance the quality improvement programs, a VAE definition was proposed. However, VAE surveillance is insensitive for identifying VAP^30^. A recent study confirmed that there was poor agreement between VAE and VAP^31^. Therefore, the outcomes of patients with VAE may be different from those with VAP. In addition, some other conditions during mechanical ventilation also cause the deterioration in gas exchange, such as pulmonary edema, acute respiratory distress syndrome, and lung atelectasis. Sepsis is also among the risk factors for the abovementioned syndromes. Therefore, the rate of VAE (118/453 [26.0%]) in our series was higher than those reported in other studies^32^. Our VAE cases include 23 (19.4%) VACs, 47 (39.8%) IVACs, and 48 (40.6%) possible VAPs. The three categories of VAE may have specific impact on outcomes; however, that is beyond the scope of this study. VACs, IVACs, and possible VAPs had been combined into one category owing to the small number of samples with VAEs. The distributions of VAE types (i.e., VAC, IVAC, and possible VAP) between the early and late VAE groups were similar (e-Table 5).

Many factors affect the outcomes of sepsis patients^33^. The severity scores (APACHE II and SOFA scores) on day 1 were comparable. Therefore, the poor outcomes in VAE group were not due to the baseline sepsis severity. Sepsis patients were admitted to the ICU primarily due to pneumonia. We tested the severity score of pneumonia, and results showed that VAE and non-VAE were comparable. The higher Charlson Comorbidity Index in the VAE group was due to the higher percentage of patients with cancer. As in our previous study, sepsis patients with underlying active cancer had higher baseline levels of plasma IL-10^9^. Although IL-10 levels were higher in the late VAE group than in the non-VAE group on day 1 and day 7, whether cancer patients were at risk of developing VAE for this reason needs further investigation. Anyway, the presence of cancer did not remain predictive for early VAE or late VAE development using multivariable logistic regression model.

Sepsis patients experience life-threatening organ dysfunction, which can be reflected on their SOFA score. The SOFA score assesses the severity of organ dysfunction in six organ systems: respiration, coagulation, liver, CV, central nervous system (CNS), and renal^34^. It can be applied to improve our understanding of the history of organ dysfunction and the interrelation between the failure of various organs^35^. Coagulation abnormalities contribute to sepsis-associated organ failure and uncontrolled inflammations^36,37^. Coagulopathy is associated with an increased incidence of early and late VAE in our study. The similar phenomenon was also observed in burn patients^38^. An elevated serum lactate concentration is a biomarker of tissue hypoperfusion during sepsis^37–39^. The elevated lactate level was noted on day 3 in the early VAE group and day 7 in the late VAE group, suggesting the relationship between tissue hypoperfusion and VAE development. The need for more fluid resuscitation, resulting in bigger positive cumulative fluid balance before the patients developed VAE, can be partially due to patients’ tissue hypoperfusion. Meanwhile, lung compliance can decrease because of pulmonary edema either due to fluid overload or sepsis progression. Furthermore, adequate oxygen delivery to peripheral tissue involves the integration of the various organs. Clear central nerve system drives the respiratory muscle to inspire air into the lung where oxygen exchange happens, and RBC takes the oxygen to the peripheral tissue. The impact of an elevated SOFA-score, coagulation disorders, and increasing lactate levels on adverse outcome is very clear. However, the relationship of the above factors with the development of VAE is less clear. Any impairment of the above organ can contribute to the development of late VAE and can be considered a risk factor (Table 4). An elevated RBC count has an inverse association with late VAE. Patients’ respiratory sub-scores will become poorer once they develop early VAE, as those who developed early VAE were excluded from the group with late VAE. This condition may explain why there was an inverse association between respiratory sub-score and late VAE development.

In addition to the above clinical data, one unique strength of our study is the performance of subpopulation analysis with immune status and cytokine study. Our results demonstrated serial immune status (HLA-DR; the levels indicate the percentage of activated serum monocyte with decreased number in immune suppression), proinflammatory cytokines (G-CSF, IL-6, TNF-α), and anti-inflammatory cytokine (IL-10) levels, which are important in patients with sepsis (Table 5). Our data showed that VAE development was associated with immune suppression (HLA-DR decreased, IL-10 elevated). Those immune dysfunctions in sepsis patients were associated with poor prognosis^10^. There were different trends by VAE status in the changes in cytokine levels (e-Table 6). Although the limited number of VAE patients enrolled in the immune study precludes further analysis, the study brings new insight into this field. The connection of VAE to VAP may include a compromised immune response^40^. By adding this phenomenon to the clinical factors, we can improve the detection of VAP.

The limitations of the study include the moderate number of patients and possible time-dependent confounding in the analyses of 28-day prognosis among the early VAE, late VAE, and non-VAE patients. We are interested in determining the influence of VAE on this episode of sepsis, which occurred upon ICU admission. However, the patients must have survived and been on the ventilator long enough to get the VAE. Therefore, the 90-day Kaplan–Meier survival curves with time zero were provided upon ICU admission. To account for immortal person time, we calculated the mortality hazard ratios between groups using Cox regression models with time-varying coefficient covariates. The number of days from intubation to VAE development was then adjusted. The Cox regression models with fixed (non-time-dependent) were also provided for reference (e-Table 7). Those models drew the same conclusion that VAE that occurred within 28 days of admission to the medical ICUs can be a predictor for poor outcomes in sepsis patients. We excluded patients who never had a period of stable or decreasing oxygenation. For those patient group, an immune dysfunction score can be used to predict the 28-day mortality^10^. In treating sepsis, strategies to reduce nosocomial infections are important^39^. In addition to predicting VAP, VAE surveillance could be used as another marker for mortality and prolonged mechanical ventilation prediction.

## Conclusions

In MV sepsis patients, after a period of improved or stable gas exchange, VAE was associated with a higher mortality, and patients with late VAE were prone to ventilation dependency. The risk factors include impaired immune response and sepsis progression with elevated SOFA score (especially in coagulation sub-score).

## Supplementary information

Supplementary information.

## Data Availability

The datasets used and/or analyzed during the current study are available from the corresponding author on reasonable request.
