# Supplementary material for: Risk factors and associated outcomes of ventilator-associated events developed in 28 days among sepsis patients admitted to intensive care unit
Source: Sci Rep. 2020 Jul 29;10:12702. doi: 10.1038/s41598-020-69731-3 (PMC7391677; doi:10.1038/s41598-020-69731-3)
Supplement: Supplementary file 1 — Supplementary information. [file 41598_2020_69731_MOESM1_ESM.pdf]

Supplemental electronic material

Risk factors and associated outcomes of ventilator-associated events developed in 28 days among sepsis patients admitted to intensive care unit

Wen-Feng Fang, Ying-Tang Fang, Chi-Han Huang, Yu-Mu Chen, Ya-Chun Chang, Chiung-Yu Lin, Kai-Yin Hung, Ya-Ting Chang, Hung-Cheng Chen, Kuo-Tung Huang, Huang-Chih Chang, Yun-Che Chen, Yi-Hsi Wang, Chin-Chou Wang, and Meng-Chih Lin

e-Table 1. Baseline and clinical characteristics of 453 patients with sepsis and comparison among the non-VAE early VAE and late VAE groups

**Demographics characteristics**	**Non-VAE (N=335)**	**Early VAE (N=33)**	**Late VAE (N=85)**	**P †**	**P§**
Age, years	68.0 ± 15.1	66.6 ± 13.8	67.6 ±14.0	0.855	0.948
BMI, kg/m^2	22.7 ± 4.9	22.8 ± 5.3	21.9 ± 4.6	0.387	0.666
Sex, male (%)	196 (58.5)	17 (51.5)	56 (65.9)	0.295	0.329
Site of suspected infection, N (%)					
Pulmonary	201 (60.0)	18 (54.5)	54 (63.5)	0.657	0.645
Intra-abdominal	23 (6.9)	5 (15.2)	6 (7.1)	0.223	0.293
Urinary tract	103 (30.7)	5 (15.2)	22 (25.9)	0.137	0.213
Bacteremia	32 (9.6)	0 (0)	9 (10.6)	0.163	0.052
Unidentified infection	27 (8.1)	2 (6.1)	5 (5.9)	0.752	0.479
APACHE II score	25.1 ± 8.7	25.2 ± 7.5	23.7 ± 8.3	0.421	0.385
CURB65	2.6 ± 0.9	2.6 ± 0.8	2.7 ± 0.9	0.586	0.691
PSI	134.1 ± 34.9	137.2 ± 32.2	141.4 ± 32.2	0.211	0.842
Charlson Comorbidity Index	2.4 ± 1.7	3.0 ± 2.0*	3.2 ± 2.2#	0.001	0.806
Coronary artery disease	84 (25.1)	6 (18.2)	24 (28.2)	0.527	0.498
Hypertension	197 (58.8)	17 (51.5)	41 (48.2)	0.182	0.944
COPD	52 (15.5)	6 (18.2)	9 (10.6)	0.442	0.551
Cancer	59 (17.8)	11 (33.3) *	32 (38.1) #	<0.001	0.840

Chronic liver disease	39 (11.6)	6 (18.2)	13 (15.3)	0.421	0.983
Diabetes mellitus	164 (49.0)	16 (48.5)	31 (36.5)	0.117	0.468
History of stroke	76 (22.7)	5 (15.2)	18 (21.2)	0.598	0.758
Chronic kidney disease	104 (31.0)	8 (24.2)	27 (31.8)	0.701	0.707
Ventilator days to VAE, d (IQR)		5 (4 – 6)	17 (12 – 28)		

†Comparison analyses among the non-VAE, early VAE, and late VAE groups using one-way analysis of variance (ANOVA).

§ The pairwise comparisons come from the ANOVA with adjustment for multiple comparisons utilizing Tukey's range test for post hoc comparisons

Categorical variables such as sex and site of suspected infection were compared using a chi-square test for categorical variables.

P§: between early and late VAE

*P < 0.05 between the non-VAE and early VAE

#P < 0.05 between the non-VAE and late VAE

e-Table 2 (a). Clinical characteristics of 453 patients with sepsis and comparison among the non-VAE, early VAE, and late VAE groups

**Clinical characteristics**	**Non-VAE (N=335)**	**Early VAE (N=33)**	**Late VAE (N=85)**	**P †**	**P §**
**Day 1**					
SOFA scores	8.9 ± 3.5	10.0 ± 3.9	9.3 ± 3.7	0.211	0.739
Respiration subscore	2.3 ± 1.2	2.3 ± 1.2	2.0 ± 1.1	0.212	0.545
Coagulation subscore	0.6 ± 1.0	1.1 ± 1.4*	0.9 ± 1.3##	0.022	0.838
Liver subscore	0.4 ± 0.8	0.5 ± 0.9	0.4 ± 0.9	0.594	0.826
CV subscore	1.1 ± 1.5	1.7 ± 1.7	1.4 ± 1.7	0.092	0.722
CNS subscore	2.9 ± 1.1	2.7 ± 1.1	3.1 ± 1.0	0.167	0.239
Renal subscore	1.3 ± 1.4	1.4 ± 1.5	1.1 ± 1.3	0.478	0.639
White blood cells, $10\times10^3/\mu L$	15.1 ± 8.3	12.8 ± 7.1	14.1 ± 9.2	0.241	0.730
Red blood cells, $10^6/\mu L$	3.6 ± 0.8	3.5 ± 0.8	3.3 ± 0.8##	0.043	0.542
C-reactive protein, mg/L	149.6 ± 117.8	129.4 ± 119.6	151.9 ± 124.7	0.687	0.688
Lactate, mmol/L	31.5 ± 31.5	33.3 ± 24.8	33.5 ± 27.0	0.870	1
Oxygenation index	8.6 ± 7.5	8.0 ± 5.1	7.1 ± 6.4	0.244	0.815
Resistance	16.5 ± 10.5	19.2 ± 12.1	17.3 ± 7.3	0.435	0.711
Compliance	51.4 ± 35.7	47.2 ± 25.3	47.5 ± 36.8	0.665	0.999
I/O, fluid balance	396.7 ±1294.8	722.8 ± 1533.9	622.1 ± 1307.6	0.186	0.926
**Day 3**					
SOFA score	7.1 ± 2.9	9.3 ± 3.9*	8.6 ± 3.6#	<0.001	0.577

Respiration subscore	1.6 ± 0.9	1.9 ± 1.1	2.0 ± 0.9#	0.001	0.897
Coagulation subscore	0.9 ± 1.1	1.6 ± 1.5**	1.2 ± 1.2##	<0.001	0.324
Liver subscore	0.4 ± 0.7	0.9 ± 1.1**	0.5 ± 0.8	0.009	0.171
CV subscore	0.4 ± 1.0	0.9 ± 1.4*	0.9 ± 1.4#	0.003	0.999
CNS subscore	2.5 ± 0.9	2.9 ± 0.8	2.7 ± 1.1	0.171	0.593
Renal subscore	1.2 ± 1.5	1.3 ± 1.6	1.2 ± 1.4	0.951	0.989
White blood cells, $10 \times 10^3/\mu L$	12.7 ± 5.9	12.3 ± 6.0	14.1 ± 9.5	0.193	0.384
Red blood cells, $10^6/\mu L$	3.4 ± 0.7	3.4 ± 0.8	3.3 ± 0.6	0.361	0.934
C-reactive protein, mg/L	119.1 ± 96.1	124.4 ± 115.3	144.8 ± 109.7	0.166	0.637
Lactate, mmol/L	16.9 ± 9.1	23.1 ± 12.0**	19.8 ± 11.2#	0.005	0.363
Oxygenation index	4.9 ± 4.6	6.8 ± 7.5	6.7 ± 5.7#	0.005	0.999
Resistance	14.8 ± 7.7	14.2 ± 5.4	15.2 ± 7.3	0.813	0.817
Compliance	56.9 ± 37.6	49.7 ± 33.8	54.0 ± 34.0#	0.051	0.850
I/O, fluid balance	400.2 ± 1271.1	694.3 ± 1310.3	895.8 ±1328.7#	0.005	0.725

e-Table 2 (b). Clinical characteristics of 453 patients with sepsis and comparison among the non-VAE, early VAE, and late VAE groups

Clinical characteristics	Non-VAE (N= 335)	Early VAE (N=33)	Late VAE (N= 85)	P†	P§
**Day 7**					
SOFA score	5.9 ± 2.9	10.7 ± 5.3*	8.8 ± 3.8	<0.001	0.022
Respiration subscore	1.5 ± 0.8	2.7 ± 1.3	2.0 ± 1.0#	<0.001	0.005
Coagulation subscore	0.6 ± 0.9	1.7 ± 1.4	1.3 ± 1.2#	<0.001	0.313
Liver subscore	0.4 ± 0.7	0.7 ± 1.0	0.5 ± 0.8	0.115	0.373
CV subscore	0.2 ± 0.6	1.1 ± 1.7	0.7 ± 1.3#	<0.001	0.212
CNS subscore	2.2 ± 1.2	3.1 ± 0.9	2.9 ± 0.9#	<0.001	0.814
Renal subscore	0.9 ± 1.3	1.5 ± 1.6	1.3 ± 1.3	0.050	0.796
White blood cells, $10\times10^3/\mu L$	12.0 ± 6.0	16.7 ± 15.4	13.0 ± 9.3	0.009	0.079
Red blood cells, $10^6/\mu L$	3.5 ± 0.6	3.3 ± 0.6	3.2 ± 6.1#	0.001	0.849
C-reactive protein, mg/L	59.2 ± 56.8	137.2 ± 116.7	116.9 ± 95.8#	<0.001	0.447
Lactate, mmol/L	13.5 ± 6.8	32.2 ± 37.6	19.5 ± 16.7#	<0.001	0.001
Oxygenation Index	4.2 ± 3.1	15.5 ± 13.6	7.2 ± 6.4#	<0.001	<0.001
Resistance	13.6 ± 5.1	20.1 ± 14.8	17.1 ± 11.9#	<0.001	0.325
Compliance	55.5 ± 30.8	36.1 ± 25.2	42.6 ± 35.1#	0.002	0.689
I/O, fluid balance	27.1 ± 1147.6	876.5 ± 1272.0	537.4 ± 1458.5#	<0.001	0.366

Oxygenation index = 100 *(F_iO_2 * mean airway pressure)/P_aO_2

I/O: input and output, fluid balance

[†]Comparison analyses among the non-VAE, early VAE, and late VAE groups using one-way analysis of variance (ANOVA).
The pairwise comparisons come from the ANOVA with adjustment for multiple comparisons utilizing Tukey's range test for post hoc comparisons

[§]: between early and late VAE

[*]$P < 0.05$ between the non-VAE and early VAE

[#]$P < 0.05$ between the non-VAE and late VAE

e-Table 3. Daily and cumulative fluid status of the sepsis patients.

		Non-VAE (n=335)			Early VAE (n=33)			Late VAE (n=85)			P†	P§
		Input	Output	I/O	Input	Output	I/O	Input	Output	I/O		
Daily fluid status	Day 1	951.6 (987.0)	1,024.8 (1,120.3)	396.7 (1,294.8)	1,271.1 (1,169.6)	923.2 (1,066.5)	722.8 (1,533.9)	1,239.5 (1,067.5)	981.2 (842.0)	622.1 (1,307.6)	0.186	0.722
	Day 3	1,027.7 (909.4)	1,507.6 (1,180.2)	400.2 (1,271.1)	1,327.8 (943.9)	1,208.4 (1,270.2)	694.3 (1,310.3)	1,327.7 (993.9)	1,291.7 (918.5)	895.8# (1,328.7)	0.005	0.459
	Day 7	677.2 (884.8)	1,506.3 (1,204.3)	27.1 (1,147.6)	1,175.7 (1,119.0)	785.7 (1,075.1)	876.5** (1,272.0)	1,144.8 (954.7)	1,418.4 (1,147.1)	537.4# (1,458.5)	<0.001	0.243
Cumulative fluid status	Days 1–2	2,174.4 (1,849.8)	2,411.2 (2,000.0)	1,124.9 (2,331.2)	3,049.9 (2,123.6)	2,081.7 (1,885.8)	1,967.5 (2,753.3)	2,796.9 (2,007.1)	2,252.5 (1,605.4)	1,661.7 (2,588.9)	0.046	0.573
	Days 1–3	3,199.7 (2,620.6)	3,920.4 (2,834.0)	1,525.2 (3,104.9)	4,377.7 (2,768.0)	3,290.2 (2,682.8)	2,661.9 (3,481.9)	4,124.6 (2,864.3)	3,544.2 (2,370.1)	2,557.6# (3,627.9)	0.009	0.888
	Days 1–4	5,142.9 (4,120.2)	5,415.5 (3,652.7)	1,855.6 (3,573.7)	7,063.6 (4,426.2)	4,329.3 (3,138.8)	3,654.7* (4,156.8)	6,656.4 (4,566.2)	4,831.9 (3,088.6)	3,257.5# (4,463.9)	0.001	0.660
	Days 1–5	5,870.0 (4,700.6)	6,981.9 (4,548.0)	1,952.1 (3,930.0)	8,178.2 (4,718.0)	5,316.1 (3,588.5)	4,380.4* (4,502.8)	7,766.6 (5,144.0)	6,248.4 (3,977.9)	3,792.2# (5,044.1)	<0.001	0.518
	Days 1–6	6,530.7 (5,187.9)	8,568.4 (5,417.5)	2,053.2 (4,263.9)	9,754.5 (5,832.4)	6,181.9 (4,064.5)	5,329.8* (5,409.9)	8,784.5 (5,654.6)	7,617.9 (4,895.4)	4,139.7# (5,524.7)	<0.001	0.293
	Days 1–7	7198.8 (5744.6)	10,050.1 (6,282.3)	2,080.3 (4,492.8)	10,930.3 (6,344.8)	6,967.7 (4,661.4)	6,206.3* (5,961.7)	9,929.3 (6,324.3)	9,013.4 (5,788.3)	4,677.1# (6,063.1)	<0.001	0.219

Comparison of I/O balance among the three groups. Data are presented as mL and mean (± SD).

[†]Comparison analyses among the non-VAE, early VAE, and late VAE groups using one-way analysis of variance (ANOVA).
The pairwise comparisons were conducted using the ANOVA with adjustment for multiple comparisons utilizing Tukey's range test for post hoc comparisons

[§] between early and late VAE

[*]$P < 0.05$ between the non-VAE and early VAE

[#]$P < 0.05$ between the non-VAE and late VAE

e-Table 4. Changes in SOFA scores and subscores

Clinical characteristics	Non-VAE	Early VAE	Late VAE	P †	P §
**Delta day 3–day 1**					
SOFA scores	−1.8 ± 2.9	−0.7 ± 3.3	−0.6 ± 2.9#	0.001	0.747
Respiration subscore	−0.7 ± 1.3	−0.5 ± 3.0	0.0 ± 1.3#	<0.001	0.055
Coagulation subscore	0.2 ± 0.8	0.5 ± 0.8	0.3 ± 1.1	0.070	0.196
Liver subscore	0.0 ± 0.5	0.3 ± 0.7*	0.1 ± 0.7	0.010	0.234
CV subscore	−0.7 ± 1.5	−0.8 ± 1.6	−0.6 ± 1.6	0.584	0.594
CNS subscore	−0.3 ± 1.1	0.1 ± 1.2	−0.4 ± 1.0	0.079	0.032
Renal subscore	−0.1 ± 0.7	−0.1 ± 0.9	0.1 ± 0.7	0.093	0.228
**Delta day 7–day 3**					
SOFA score	−1.3 ± 2.2	1.6 ± 2.9*	0.2 ± 2.8#	<0.001	0.039
Respiration subscore	−0.1 ± 1.0	0.7 ± 1.0*	0.0 ± 1.1	0.004	0.012§§
Coagulation subscore	−0.3 ± 0.8	0.1 ± 0.8	0.1 ± 1.0#	0.001	0.943
Liver subscore	−0.1 ± 0.6	0.2 ± 0.8	0.0 ± 0.5	0.324	0.407
CV subscore	−0.3 ± 1.0	0.4 ± 1.6	−0.1 ± 1.5	0.108	0.360
CNS subscore	−0.4 ± 1.1	0.3 ± 1.0*	0.3 ± 1.2#	<0.001	0.664
Renal subscore	−0.3 ± 0.7	0.3 ± 0.8*	0.0 ± 0.8#	<0.001	0.123

**Delta day 7–day 1**					
SOFA score	−3.1±3.4	0.8±5.1*	−0.4±3.5#	<0.001	0.408
Respiration subscore	−0.8±1.4	0.2±1.4*	−0.1±1.3#	<0.001	0.384
Coagulation subscore	0.0±1.1	0.6±1.2*	0.5±1.4#	<0.001	0.603
Liver subscore	−0.1±0.7	0.5±0.8*	0.1±0.6	0.001	0.016§§
CV subscore	−0.9±1.6	−0.6±2.2	−0.7±1.8	0.287	0.959
CNS subscore	−0.7±1.4	0.3±1.3*	−0.2±1.1#	<0.001	0.049
Renal subscore	−0.4±0.9	0.1±1.3*	0.1±1.0#	<0.001	0.930

Delta day 3–day 1: the change in values from day 1 to day 3

Delta day 7–day 3: the change in values from day 3 to day 7

Delta day 7–day 1: the change in values from day 1 to day 7

P† : Comparison analyses among the non-VAE, early VAE, and late VAE groups using Kruskal-Wallis as a non-parametric alternative to the ANOVA for non-normally distributed continuous variables.

Post hoc pairwise comparisons

*P < 0.05, between the non VAE and early VAE

#P< 0.05, between the non-VAE and late VAE

§§P< 0.05, between early and late VAE

P§: Comparison analyses between the early VAE and late VAE groups using Mann-Whitney U test

e-Table 5. Distribution of VAE types

	Early VAE	Late VAE	
VAC	9	14	23
IVAC	13	34	47
Possible VAP	11	37	48
	33	85	

e-Table 6. Changes in cytokine parameters

**Delta day 3–day 1**	**Non-VAE**	**Early VAE**	**Late VAE**	**P †**	**P §**
HLA-DR expression, %	2.4 ± 11.4	13.7 ± 22.0	−0.4 ± 18.3	NA	0.144
G-CSF, pg/mL	504.2 ± 655.4	NA	2323.5	NA	NA
IL-10, pg/mL	−21.8 ± 80.5	186.5 ± 578.3	−94.8 ± 314.1	NA	0.556
IL-6, pg/mL	−69.3 ± 219.6	240.7 ± 624.5	−1231.7 ± 4418.8	NA	0.845
TNF-α, pg/mL	−9.9 ± 48.9	25.1 ± 84.7	−69.4 ± 198.3	NA	0.292
**Delta day 7–day 3**	**Non-VAE**	**Early VAE**	**Late VAE**	**P †**	**P §**
HLA-DR expression, %	4.6 ± 10.3	3.7 ± 4.9	−1.3 ± 10.0	NA	0.437
G-CSF, pg/mL	−16.7 ± 55.1	NA	7.9	NA	NA
IL-10, pg/mL	7.9 ± 73.5	0.7 ± 16.3	−9.3 ± 63.3	NA	0.820
IL-6, pg/mL	−26.0 ± 173.0	8.3 ± 29.8	−57.4 ± 323.7	NA	0.291
TNF-α, pg/mL	−3.5 ± 33.2	−5.3 ± 9.1	1.7 ± 19.5	NA	0.616
**Delta day 7–day 1**	**Non-VAE**	**Early VAE**	**Late VAE**	**P †**	**P §**

HLA-DR expression, %	6.9 ± 12.8	16.2 ± 12.4	−1.7 ± 16.7	NA	0.041
G-CSF, pg/mL	−19.4 ± 56.2	NA	9.1	NA	NA
IL-10, pg/mL	−17.4 ± 119.9	−7.0 ± 13.2	−101.5 334.7	NA	0.820
IL-6, pg/mL	−101.5 ± 301.9	−20.5 ± 44.4	−1392.4 ± 4588.5	NA	0.682
TNF-α, pg/mL	−13.0 ± 50.7	−6.1 ± 11.0	−13.6 ± 44.8	NA	0.892

Delta day 3–day 1: the change in values from day 1 to day 3

Delta day 7–day 3: the change in values from day 3 to day 7

Delta day 7–day 1: the change in values from day 1 to day 7

***P*†** : Comparison analyses among the non-VAE, early VAE, and late VAE groups using Kruskal-Wallis as a non-parametric alternative to the ANOVA for non-normally distributed continuous variables

*P < 0.05, **P < 0.01 between the non VAE and early VAE

#P< 0.05, ##P < 0.01 between the non-VAE and late VAE

P §: Comparison analyses between the early VAE and late VAE groups using Mann-Whitney U test

NA: data not available due to the limited number of cases

e-Table 7. Hazard ratios between groups using Cox regression models with time-varying coefficient or fixed (non-time-dependent) covariates

(A) 90-d Cox regression model with time-varying coefficient (time to VAE development after intubation)

Hazard ratios with 95% confidence interval

Factors	Non-VAE	Early VAE	Late VAE
Non VAE	1	2.643 (1.321-5.287)	2.457 (1.524-3.961)

(B) 90-d Cox Regression model with fixed (non-time-dependent) covariates

Hazard ratios with 95% confidence interval

Factors	Non-VAE	Early VAE	Late VAE
Non VAE	1	8.478 (5.459–13.166)	4.799 (3.463–6.650)

(C) Comparison between groups (P value)

	Non-VAE	Early VAE	Late VAE
Non-VAE	1	*<0.001**	*<0.001**
Early VAE	**0.006#**	1	*<0.001**
Late VAE	**<0.001#**	**0.769#**	1

*: Fixed (non-time-dependent) covariates

#: with time-varying coefficient (time to VAE development after intubation)

e-Figure 1. Ninety-day survival curves between non-VAE and VAE group

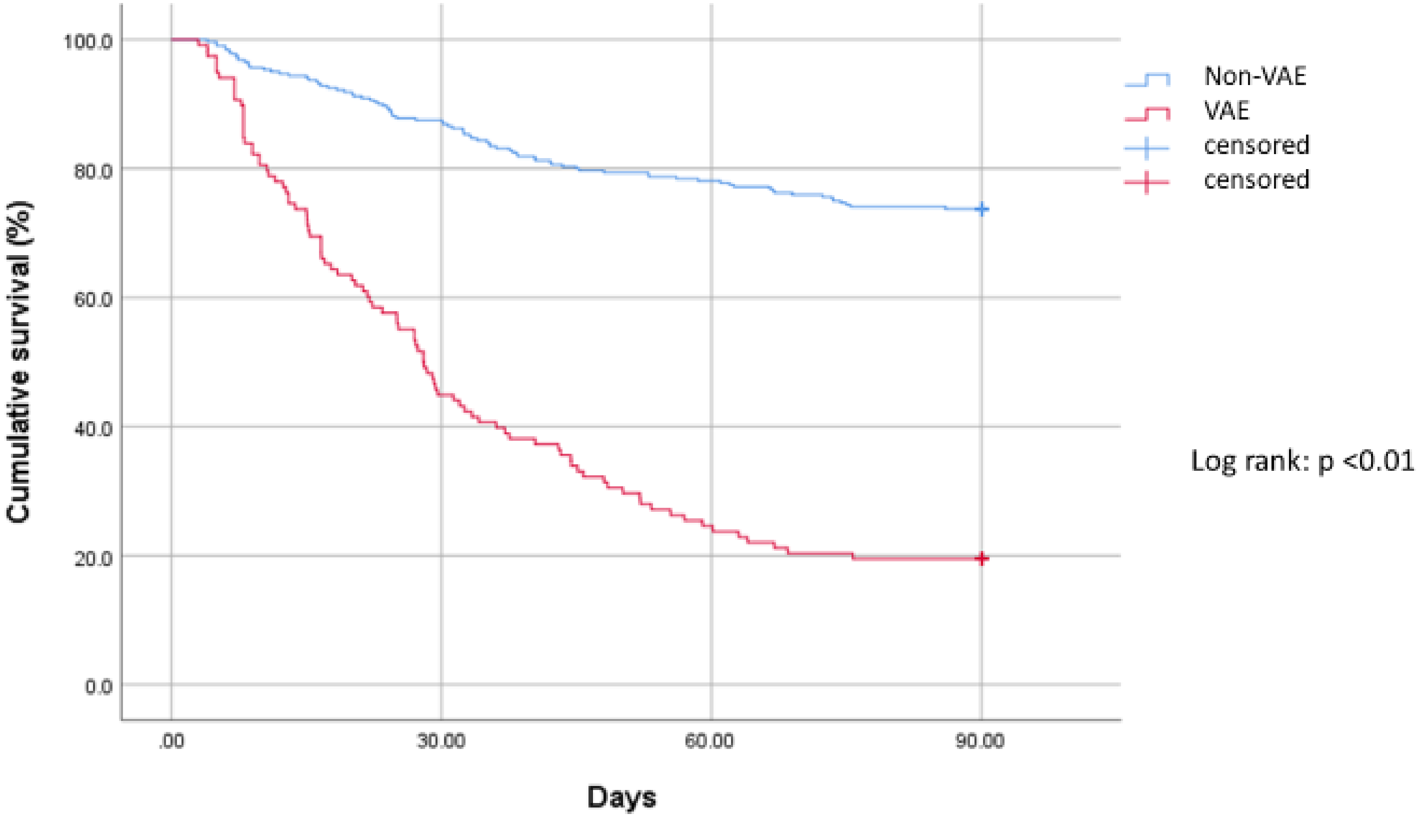

e-Figure 2. Ninety-day survival curves between patients with immune data group and without immune data group

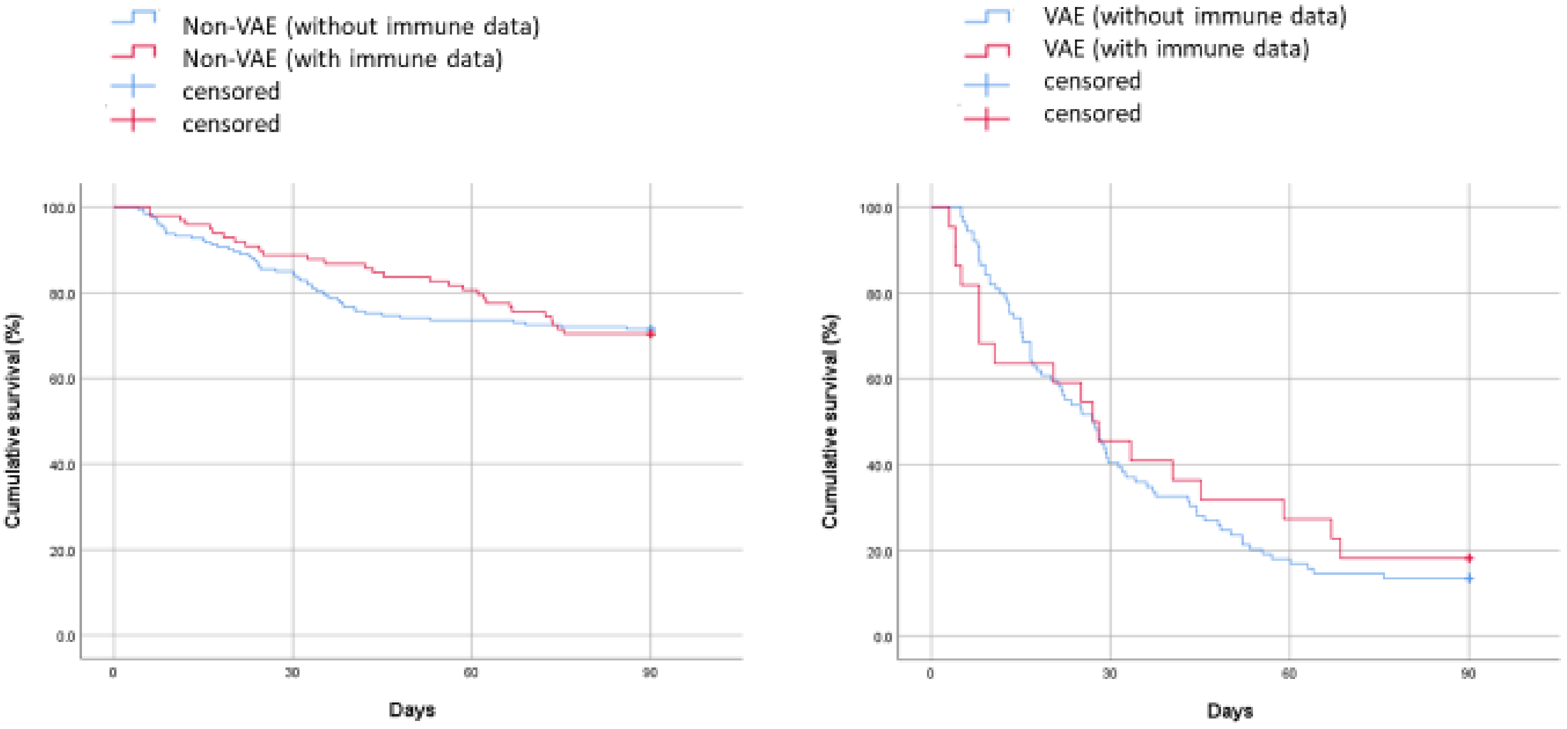

Log rank: p=0.690

Log rank: p=0.944
